# Supplementary material for: A blind benchmark of analysis tools to infer kinetic rate constants from single-molecule FRET trajectories
Source: Nat Commun. 2022 Sep 14;13:5402. doi: 10.1038/s41467-022-33023-3 (PMC9474500; doi:10.1038/s41467-022-33023-3)
Supplement: Supplementary file 2 — Reporting Summary [file 41467_2022_33023_MOESM2_ESM.pdf]

nature portfolio

Corresponding author(s): Markus Götz, Sonja Schmid

Last updated by author(s): Aug 24, 2022

Reporting Summary

Nature Portfolio wishes to improve the reproducibility of the work that we publish. This form provides structure for consistency and transparency in reporting. For further information on Nature Portfolio policies, see our Editorial Policies and the Editorial Policy Checklist.

Statistics

For all statistical analyses, confirm that the following items are present in the figure legend, table legend, main text, or Methods section.

n/a	Confirmed	
☐	☒	The exact sample size (*n*) for each experimental group/condition, given as a discrete number and unit of measurement
☐	☒	A statement on whether measurements were taken from distinct samples or whether the same sample was measured repeatedly
☒	☐	The statistical test(s) used AND whether they are one- or two-sided<br>*Only common tests should be described solely by name; describe more complex techniques in the Methods section.*
☒	☐	A description of all covariates tested
☒	☐	A description of any assumptions or corrections, such as tests of normality and adjustment for multiple comparisons
☐	☒	A full description of the statistical parameters including central tendency (e.g. means) or other basic estimates (e.g. regression coefficient) AND variation (e.g. standard deviation) or associated estimates of uncertainty (e.g. confidence intervals)
☒	☐	For null hypothesis testing, the test statistic (e.g. *F*, *t*, *r*) with confidence intervals, effect sizes, degrees of freedom and *P* value noted<br>*Give P values as exact values whenever suitable.*
☒	☐	For Bayesian analysis, information on the choice of priors and Markov chain Monte Carlo settings
☒	☐	For hierarchical and complex designs, identification of the appropriate level for tests and full reporting of outcomes
☒	☐	Estimates of effect sizes (e.g. Cohen's *d*, Pearson's *r*), indicating how they were calculated

Our web collection on statistics for biologists contains articles on many of the points above.

Software and code

Policy information about availability of computer code

Data collection	The scripts and parameters necessary to generate the simulated datasets (Figs 2, 3, and 4) are available for download at zenodo https://doi.org/10.5281/zenodo.5701310 (version v0).
Data analysis	All software tools are available: Pomegranate v0.0.1 at https://github.com/hatzakislab/DeepFRET-GUI ; Tracy v4.4.8 upon request as it is being replaced by a new program for multi-color analysis (contact: Don C. Lamb [d.lamb@lmu.de], requests will be addressed as soon as possible, typically within 1 week); FRETboard v0.0.3 at https://github.com/cvdelannoy/FRETboard ; Hidden-Markury v0.0.1 at https://github.com/ChristianGebhardt/Hidden-Markury ; SMACKS v1.4 at https://github.com/sciSonja/SMACKS ; Correlation v0.1b at https://doi.org/10.5281/zenodo.5512005 ; Edge finding (CK and k-means) v0.0.1 at https://www.physics.ncsu.edu/weninger/KinSoft.html ; Step finding v0.0.2 at https://github.com/SMB-Lab/PyStepFinder ; StaSI v0.0.1 at https://github.com/LandesLab/StaSI ; MASH-FRET v.1.3.2 (bootstrap and probabilistic) at https://github.com/RNA-FRETools/MASH-FRET ; postFRET v4.0 at https://github.com/nkchenjx/postFRET .

For manuscripts utilizing custom algorithms or software that are central to the research but not yet described in published literature, software must be made available to editors and reviewers. We strongly encourage code deposition in a community repository (e.g. GitHub). See the Nature Portfolio guidelines for submitting code & software for further information.

Data

Policy information about availability of data

All manuscripts must include a data availability statement. This statement should provide the following information, where applicable:

- Accession codes, unique identifiers, or web links for publicly available datasets
- A description of any restrictions on data availability
- For clinical datasets or third party data, please ensure that the statement adheres to our policy

The simulated and experimental smFRET data used in this study are available at www.kinsoftchallenge.com and zenodo https://doi.org/10.5281/zenodo.5701310 . All inferred results are provided in the Supplementary Data files. Supplementary figures, notes, and methods are provided in the Supplementary Information file. Source data are provided with this paper.

Field-specific reporting

Please select the one below that is the best fit for your research. If you are not sure, read the appropriate sections before making your selection.

- [x] Life sciences
- [ ] Behavioural & social sciences
- [ ] Ecological, evolutionary & environmental sciences

For a reference copy of the document with all sections, see nature.com/documents/nr-reporting-summary-flat.pdf

Life sciences study design

All studies must disclose on these points even when the disclosure is negative.

Sample size	Sample sizes of experimental datasets: data were used as reported in the cited publications. No adjustment to the sample size was made. Sample sizes of simulated datasets were designed to be similar to the ones of experimental datasets (in terms of number of traces and total datapoints), which are typical in the field. Supplementary Figure 3 shows that the sample size is sufficient for good reproducibility.
Data exclusions	No data was excluded.
Replication	Does not apply. No new experimental data are presented in this study.
Randomization	Does not apply. In this blind study, all tools worked on exactly the same datasets to ensure comparability of the results.
Blinding	This benchmark was conducted as a blind study. In a few cases (clearly marked with a dagger in all Figures), the ground truth and/or participant results were already known, when the inferred rate models were reported.

Reporting for specific materials, systems and methods

We require information from authors about some types of materials, experimental systems and methods used in many studies. Here, indicate whether each material, system or method listed is relevant to your study. If you are not sure if a list item applies to your research, read the appropriate section before selecting a response.

Materials & experimental systems

n/a	Involved in the study
[x]	[ ] Antibodies
[x]	[ ] Eukaryotic cell lines
[x]	[ ] Palaeontology and archaeology
[x]	[ ] Animals and other organisms
[x]	[ ] Human research participants
[x]	[ ] Clinical data
[x]	[ ] Dual use research of concern

Methods

n/a	Involved in the study
[x]	[ ] ChIP-seq
[x]	[ ] Flow cytometry
[x]	[ ] MRI-based neuroimaging
