# Supplementary material for: A blind benchmark of analysis tools to infer kinetic rate constants from single-molecule FRET trajectories
Source: Nat Commun. 2022 Sep 14;13:5402. doi: 10.1038/s41467-022-33023-3 (PMC9474500; doi:10.1038/s41467-022-33023-3)
Supplement: Supplementary file 17 — Description of Additional Supplementary Files [file 41467_2022_33023_MOESM17_ESM.pdf]

Legends for the Supplementary Data files

Title: **Supplementary Data 1**
Description: Configuration file with the simulation parameters for the data shown in Fig. 2b, c, d.

Title: **Supplementary Data 2**
Description: Configuration file with the simulation parameters for the data shown in Fig. 3.

Title: **Supplementary Data 3**
Description: Configuration file with the simulation parameters for the data shown in Fig. 4.

Title: **Supplementary Data 4**
Description: Readme file for the xlsx files containing the submitted results of the participating labs.

Title: **Supplementary Data 5**
Description: Inferred FRET efficiencies and rate constants for each lab; corresponding to the data shown in Fig. 2c, d.

Title: **Supplementary Data 6**
Description: Inferred FRET efficiencies and rate constants for each lab; corresponding to the data shown in Fig. 2f, g.

Title: **Supplementary Data 7**
Description: Inferred FRET efficiencies and rate constants for each lab; corresponding to the data shown in Supp. Fig. 2b, c.

Title: **Supplementary Data 8**
Description: Inferred FRET efficiencies and rate constants for each lab; corresponding to the data shown in Fig. 3.

Title: **Supplementary Data 9**
Description: Inferred FRET efficiencies and rate constants for each lab; corresponding to the data shown in Fig. 4.

Title: **Supplementary Data 10**
Description: Inferred FRET efficiencies and rate constants for each lab; corresponding to the data shown in Fig. 5b, c and Supp. Figs. 4, 5, and 6. The suffix “_FS_<n>” indicates the number of FRET states for each submission.

Title: **Supplementary Data 11**

Description: Inferred FRET efficiencies and rate constants for each lab; corresponding to the data shown in Fig. 5e, f and Supp. Figs. 4, 5, and 6. The suffix “_FS_<n>” indicates the number of FRET states for each submission.

Title: **Supplementary Data 12**

Description: Inferred FRET efficiencies and rate constants for each lab; corresponding to the data shown in Fig. 5h, i and Supp. Figs. 4, 5, and 6. The suffix “_FS_<n>” indicates the number of FRET states for each submission.

Title: **Supplementary Data 13**

Description: Updated uncertainties of the results shown in Figs. 2 and 3 reported by the tool *FRETboard* after the ground truth was released.

Title: **Supplementary Data 14**

Description: Updated uncertainties of the results shown in Figs. 2, 3, 4, and Supp. Fig. 2b,c reported by the tool *StepFinding* after the ground truth was released.
